# Assessment of feto-maternal hemorrhage among rhesus D negative pregnant mothers using the kleihauer-betke test (KBT) and flow cytometry (FCM) in Addis Ababa, Ethiopia

**DOI:** 10.1186/1471-2393-14-358

**Published:** 2014-11-07

**Authors:** Fekadu Urgessa, Aster Tsegaye, Yirgu Gebrehiwot, Asaye Birhanu

**Affiliations:** Department of Medical Laboratory Science, Haramaya University, Harar, Ethiopia; Department of Medical Laboratory Science, Addis Ababa University, Addis Ababa, Ethiopia; Department of Obstetrics and Gynecology, Addis Ababa University, Addis Ababa, Ethiopia

**Keywords:** Fetomaternal hemorrhage, RhIG, Flow cytometry and Kleihauer-Betke/Acid elution test

## Abstract

**Background:**

This study aimed to assess fetomaternal hemorrhage (FMH) among RhD negative pregnant mothers using two techniques, Kleihauer-Betke (KBT) and Flow cytometry (FCM). To determine if patient-specific doses of prophylactic anti-D warrant further investigation in Ethiopia and wider Africa.

**Methods:**

Hospital- based cross-sectional study was conducted among 75 RhD negative pregnant mothers using convenient sampling technique.

**Result:**

FMH has been detected in 52% and 60% by KBT and FCM techniques, respectively. The volume of FMH quantified in the majority of the cases (92.5% and 87%) was <10 mL fetal blood while >30 mL in 1.3% (1/75) and 2.7% (2/75) as calculated by KBT and FCM, respectively. The FMH calculated by the two methods have good correlation; r = 0.828 (p = 0.000) for categorized and r = 0.897 (p = 0.000) for continuous values and the agreement between the FCM and KBT was moderate with kappa (κ) value of 0.53 (p = 0.000).

**Conclusion:**

Most of FMH calculated (<10 mL) could have been neutralized by lower doses which might have lower costs than administering 300 μg dose which is currently in practice in our country for affording mothers. Besides, it also showed that the volume of FMH was >30 mL in 1.3% and 2.7% of the cases as calculated by KBT and FCM, respectively, which need more than 300 μg dose RhIG for neutralization. Further investigation into the cost- effectiveness and scalability of patient- specific dosing of prophylactic anti-D appears warranted.

## Background

Alloimmune hemolytic diseases of the fetus and newborns (HDF/N) results from the destruction of red cells by maternal immunoglobulin (IgG) antibodies that gain access to the fetal circulation during gestation. The most serious form of HDFN is caused by maternal alloantibodies directed against the D antigen of the Rh blood group system due to the high immunogenicity of D antigen. RhD HDFN in Rh-negative women can be prevented if the appropriate dose of prophylactic anti-D (RhIG) is given at the appropriate time [1–6].

All D-negative women who deliver a D-positive fetus should receive at least a single 300-μg dose of RhIG within 72 hours of delivery. In addition, a maternal sample should be obtained approximately 1 hour after delivery and tested for evidence of a FMH in excess of 30 mL of fetal blood. Approximately 17% of Rh D–negative women who deliver Rh D–positive fetus become alloimmunized if RhIG is not administered appropriately. RhIG prophylaxis has reduced the overall risk of Rh immunization from 13.2% to 0.2%, and testing for large FMH has further decreased the risk to 0.14%. Hence, RhD immunization may be further reduced by strict compliance to guidelines concerning determination of FMH and accordingly adjusted RhIG or by routine administration of extra RhIG after a non-spontaneous delivery and/or a complicated or prolonged third stage of labour [7–11].

The true incidence of clinically significant fetomaternal hemorrhage is probably underreported, as an unselected population has not been screened before delivery [11]. The possibility to accurately detect FMH and precisely determine its volume would enable more effective and less costly prevention of RhD alloimmunization. Anti-D immunoglobulin could be administered only in indicated cases and only in doses essentially necessary for prevention of RhD alloimmunization [12].

Rh alloimmunization remains a major factor responsible for perinatal morbidity and it may result in the compromise of the woman’s obstetric care due to the unaffordability of RhIG in Sub-Saharan Africa. Hence, there is an urgent need for the implementation of universal access to appropriate doses of prophylactic anti-D for the Rh-negative pregnant population. There is also a need for the availability of FMH measurements following potentially sensitizing events in Africa [13].

In most health facilities in Ethiopia, quantification of FMH which could have been significant to ensure a sufficient dose of RhIG given for RhD negative mother is not common. The common clinical practice is to administer 300 μg doses to every affording unsensitized woman. This study was, therefore, carried out in the light of the needs described above and due to absence of published study on feto-maternal hemorrhage in the study area and in the country at large.

## Methods

### Samples

A total of 86 blood samples were collected in EDTA test tubes from RhD negative mothers during perinatal and postnatal periods. Our study participants were mothers who came for delivery and other obstetric and gynecological complications (Stillbirth, Abortion or Miscarriage) at Tikur Anbessa, St. Paul and Gandhi Hospitals from March 15 to May 15, 2013. Eleven samples were excluded because either the fetus blood group was known to be RhD negative or unknown. The study protocol was approved by Ethnical and Research committees of the department of Medical Laboratory Science, Addis Ababa University (Ref. MLS/056//2012), and reviewed by all Hospitals research and ethics committee. Prior to enrolment written or Oral informed consent was obtained from the participants based on literacy of the participants.

### KBT method of FMH determination

#### KBT slide preparation

Two hundred microliters of each sample was mixed with 200 μL PBS. Conventional blood films were prepared using the prepared sample and air dried at room temperature. Blood film fixed with 80% alcohol for 5 minute and air dried after fixation. Slides have been flooded with elusion solution at room temperature for 20 sec and rinsed in distilled water, air dried. Stained with 1% eosin for 2 minute and rinsed in tap water and air dried. Examination of the slide followed.

### KBT FMH calculation

When dry blood films are fixed and then immersed in an acid buffer solution, HbA is denatured and eluted, leaving red-cell ghosts. Red cells containing HbF are resistant to the acid and the hemoglobin can be stained; these cells stand out in a sea of ghost maternal cells. Fetal erythrocytes were counted in 2000 background red cells using a ×40 objective. Adult red blood cells that contained small amounts of HbF were distinguished from fetal blood cells by intensity and intracellular distribution of the pink staining. The following formula and assumptions were used to calculate FMH;

The maternal red cell volume is 1800 mL

Fetal cells are 22% larger than maternal cells

Only 92% of fetal cells stain darkly

**The fetal bleed should be calculated as follows:**

Or can be simplified to: number of fetal cells per high power field × 2400 number of maternal cells per high power field [14].

### FCM method of FMH determination

#### FCM protocol

The tests used for detection of fetal cells target Rhesus D using fluorochrome-conjugated anti-D (Santa Cruz Biotechnology, Inc., Heidelberg, Germany). It quantifies the dose of RhIG provided for RhD negative pregnant women. The calculation method of the test is as follows;

This is calculated using the formula, which assumes that:

The maternal red cell volume is 1800 mL

Fetal cells are 22% larger than maternal cells

**The fetal bleed should be calculated as follows:**

**OR can be simplified to: percentage fetal cells × 18 × 1.22**

### FCM analysis

Flow cytometric analyses was performed on a fluorescence-activated cell sorter (FACSCalibur, BD company, USA), and sample acquisition was performed on 500,000 cells at a flow rate of 5,000 events per second. Gates were set to include RBC but to exclude auto fluorescent nucleated cells. Data analysis was performed with the cell quest software (Becton Dickinson, USA). The region of analysis for fetal RBC was determined by using the positive control samples containing fetal RBC from cord blood [3, 5, 14].

### Data management and statistical analysis

The data was entered into and analyzed by SPSS version 16.0. Association of different demographic, clinical and laboratory parameters to dependent variables was computed using multiple regression analysis at 0.05 level of significance and one-way ANOVA to explore the impact of independent variables on FMH. The Pearson correlation coefficient analysis and Student’s t-test (paired, two-tailed with a significance level of *p* = 0.05) were used for the comparison of KBT and FCM results of our samples. A kappa value was used to compare the agreement of two methods.

## Result

### Socio demographic and clinical characteristics

A total of 86 blood samples were collected from RhD negative mothers during perinatal and postnatal periods. Eleven samples were excluded because either the fetus blood group was RhD negative or unknown. The majority of the mothers, 62.7% (47/75), were in the age group 21–30 years, 75% (56/75) had previous history of pregnancy and 72% (54/75) had multiple delivery (2–4 pregnancy), but 49.3% (37/75) and 28% (21/75) of the participants did not take RhIG during their previous and current pregnancy, respectively. Eighty percent (80%) (60/75) of the study participants were on antenatal care follow up during pregnancy and knew their blood group, but above 65% of them didn’t know their partner’s blood group and more than 60% (45/75) of participants’ partners’ blood group was not known. Our data also indicated 42.7% (32/75) of the participants had a gestational age of >37 weeks and only 9.3% were <12 weeks. Pregnancy outcome was successful in 77.3% of them of whom 57.3% delivered live child with >2500 g birth weight and 64% (48/75) had normal delivery (spontaneous vaginal delivery).

### Feto-maternal hemorrhage (FMH) quantification

#### FMH Calculated by Kleihauer-Betke test (KBT)

Using the KBT method, 52% (39/75) of our participants had fetal whole blood in their blood circulation during postnatal or other procedures. The amount of fetal whole blood calculated was 0.95-38 mL. Three samples had excess FMH (>30 ml) and were excluded from the statistical analysis. The mean of FMH calculated by this method after exclusion of the three extreme cases were 1.4 ± 1.8 (mean ± standard deviation). As depicted in (Table 1), the amount of fetal whole blood detected in more than 70% (28/39) of the mothers was <4 ml whereas in 22.5% (9/39), 3.25% (1/39) and 3.25% (1/39) of them the amount was 4-10 ml, 10–30 and >30 ml, respectively. For this method large FMH (>30 ml) calculated was only in one case 3.25% (1/39), but the prevalence of large FMH from all participants was 1.3% ( 1/75).

**Table 1:** **Quantification of FMH (Categorized) by KBT and FCM among RhD negative mothers (n = 75)**

Method	Amount of FMH	Frequency	Percent
**KBT**	Negative	36	48.0
	<4 ml	28	37.3
	4-10 ml	9	12.0
	10-30 ml	1	1.3
	>30 ml	1	1.3
	Total	75	100.0
**FCM**	Negative	30	40.0
	<4 ml	19	25.3
	4-10 ml	20	26.7
	10-30 ml	4	5.3
	>30	2	2.7
	Total	75	100.0

#### FMH calculated by flow cytometry (FCM)

With this method 60% (45/75) of the mothers had fetal red cells. The amount of fetal whole blood calculated was 0.74 -35.7 mL and had little variation with fetal whole blood calculated by the KBT method. The mean ± SD of FMH calculated by this method after exclusion of the three extreme cases were 3.3 ± 3.6. The volume of fetal whole blood calculated with this method in the majority of the mothers, 87% (39/45), was <10 mL whereas only in 8.9% (4/45) and 4.4% (2/45) the fetal whole blood reached 10-30 ml and >30 ml, respectively (Table 1). But from all participants, the incidence of large FMH was 2.7% (2/75) (Table 1).

### Doses of anti-D needed

In this study, the amount of FMH calculated ranged from 0.95 to 38 mL for KBT method and slightly lower (from 0.74 to 35.7 mL) when using FCM. Thus, to neutralize these amounts of fetal blood there is a need for 50 to 300 μg or more anti-D Ig. As it is shown in Table 1, the FMH calculated in the majority of the participants (37.3% for KBT and 25.3% for FCM) was <4 mL which is expected to be neutralized by 50 μg RhIG since 20-25 μg can neutralizes around 2 ml of fetal whole blood. Those with FMH of 4-10 mL (12% for KBT and 26.7% for FCM) might have needed around 100 μg RhIG prophylaxis while those with 10-30 mL and >30 m (around 2.6% for KBT and 8% for FCM) need other doses that would be >100 μg but also greater amount of RhIG depending on specific FMH calculated.

### Comparison of KBT and FCM methods

FMH calculated by the two methods have good correlation where r = 0.828 (p = 0.000) for the categorized values and r = 0.897 (p = 000) for the continuous values with a respective mean ± SD of 1.4 ± 1.8 and 3.3 ± 3.6, for KBT and FCM methods; besides, the agreement between two methods was moderate (κ = 0.53; 95% CI, 0.000 to 0.039, p = 0.000) (Table 2).

**Table 2:** **Symmetric measures between KBT and FCM method in 75 RhD negative mothers (n = 75)**

		Value	Asymp. Std. Error	Approx. T	P value
Interval by interval	Pearson’s R	.870	.035	15.074	.000
Ordinal by ordinal	Spearman Correlation	.859	.045	14.313	.000
Measure of agreement	Kappa	.530	.069	7.332	.000
N of valid cases		75			

Student’s t-test (paired, two -tailed with a significance level of *p* = 0.05) for comparing amount of fetal blood calculated by FCM and KBT (continuous) was performed. Accordingly, there was a significant difference between the size of FMH quantified using the two methods (p = 0.000, t = -7.250). FCM has detected FMH greater than KBT with 1.8 mL ± 2.16 mL fetal blood (mean ± SD) (Table 3).

**Figure 1:**
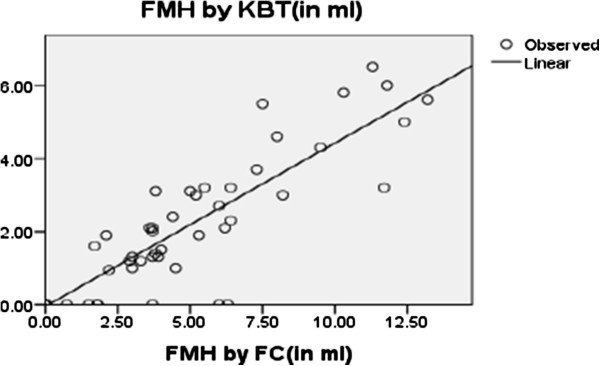
**The result of KBT method compared with FCM by linear regression analysis/continuous.**

Linear regression performed to see the linear relation between the FCM and KBT methods showed that the two methods have good linearity (Figure 1).

**Table 3:** **T-test for comparing KBT and FCM determined FMH in mL (Paired Samples Test) among RhD negative mothers (n = 75)**

	Paired differences							
				95% confidence interval of the difference				
	Mean	Std. deviation	Std. error mean	Lower	Upper	t	df	P value
FMH by KBT (in ml) - FMH by FCM (in ml)	-1.8	2.16	.2542	-2.35	-1.33	-7.250	71	.000

### Risk factors

**Figure 2:**
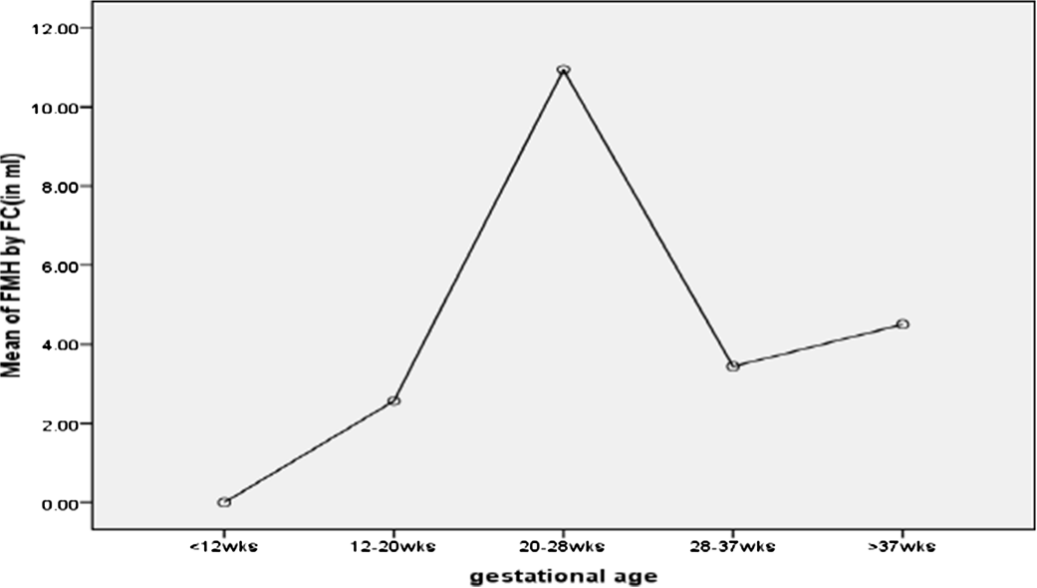
**Mean plot for mean of FMH by FCM (continuous) versus gestational age.**

All independent variables had no association with FMH calculated with both methods. However, a one-way ANOVA between-groups analysis of variance was performed to explore the impact of gestational age on levels of FMH (by FCM, continuous), as measured by Life Orientation Test (LOT). Subjects were divided into five groups according to their gestational age (Group 1; <12 weeks 2; 12–20 weeks 3; 20–28 weeks, 4; 28–37 and 5 ; > 37 weeks). Post-hoc comparisons using the Turkey HSD test indicated that the mean score for Group3 (20–28 weeks) (M = 10.9 SD = 11.1) was significantly different from all groups except with Group 5. Group1 (M = 0 SD = 0), group2 (M 2.5 = SD = 2.4), Group4 (M = 3.4 SD = 4.9) and Group5 (M = 4.5 SD = 6). The means plot provides an easy way to compare the means scores for different groups (Figure 2).

## Discussion

### Prevalence of FMH detected

Studies have shown that some degree of fetal-maternal transplacental hemorrhage occurs in 75% of all pregnancies. This phenomenon is not dangerous to the fetus unless there is incompatibility between the mother and her fetus with respect to the D antigen of the red blood cells. FMH occurs in 3% of pregnancies in the first trimester, 12% in the second trimester, 45% in the third trimester, and 64 to 100% after delivery [1, 7]. Our result has shown that FMH occurs in 52% and 60% of our participants after delivery and other procedures when employing KBT and FCM methods, respectively.

The total volume of fetal cells in the maternal circulation is usually small and does not exceed 0.1 to 0.25 ml in most cases, but large-volume FMH occurs less often, with more than 15 ml of fetal red cells (approximately 30 ml whole blood) detected at a rate of 1.6% after cesarean section or complicated vaginal delivery and 0.7% after spontaneous vaginal delivery [1]. Our result has indicated that around 92.5% and 87% of FMH calculated were <10 ml of fetal whole blood (<5 ml fetal RBC) whereas the remaining 7.5% and 13% were >10 ml of fetal whole blood (>5 ml fetal RBC) for KBT and FCM methods, respectively.

This result was inconsistent with the result revealed by Augustson *et al.* in which they concluded that 90.4% (4651/5148) of the women had FMH volume of 1.0 mL or less of Rh D-positive red cells, and 98.5% (5072/5148) had a volume of less than 2.5 mL. Only 0.4% of the cases had an FMH volume of 6.0 mL or greater (range, 6.0–92.4 mL) [15]. The variation of the result might be due to small sample size we used in contrast to Augustson *et al*.

In this study FMH of >30 mL was observed in 1.3% and 2.7% of the mothers by KBT and FCM methods, respectively. It was inconsistent with Johnson e*t al.* findings that stated in only 0.5% of deliveries FMH exceeds 25 mL [16].

### Comparison of KBT and FCM methods

FCM may be helpful for the accurate quantitation and management of patients with large FMH and in cases where the presence of maternal hemoglobin F containing cells renders the KBT technique inaccurate. While a well standardized KBT is appropriate as a screening test for FMH, studies to assess the role of FCM for detecting FMH are warranted [16]. Our result has shown that FMH calculated by FCM and KBT have good correlation for categorized values (r = 0.828, p < 0.005) as well as for continuous values (r = 0.897, p = 0.000). This finding was consistent with the study conducted by Pastoret *et al.* that revealed a good correlation between FCM and KBT (r = 0.87) [17]. In contrast to this, a study conducted by Johnson *et al.* verified the correlation between KBT and FCM results was poor. In 38 (88%) cases the size of FMH quantitated by FCM was lower than that estimated using the KBT technique. In 13 (30%) cases no Rh D immunoglobulin positive cells were detected by FCM [16].

The agreement between the two methods was moderate with the kappa value (κ = 0.53; 95% CI, 000 to 0.039 p = 0.000) that show the two methods have agreement for calculating RhD + ve FMH. Our result was consistent with a study conducted by Savithrisowmya *et al.* that verified the volume of post-delivery FMH estimated by KBT and FCM correlated well (r = 0.75; ICC α = 0.73) [18]. However, less consistent with the study conducted by Pelikan *et al.* which showed that the agreement between the manual KBT and FCM was fair with a weighted k, 0.40; 95% CI, 0.15-0.66 and correlation (r) of 0.69 [19].

### Dose requirements of prophylactic RhIG

The possibility to accurately detect FMH and precisely determine its volume would enable more effective and less costly prevention of RhD alloimmunization. RhIG could be administered only in indicated cases and only in doses essentially necessary for prevention of RhD alloimmunization [12]. As indicated the findings of our study verified FMH calculated ranges from 0.95 to 38 mL and from 0.74 to 35.7 mL with means of 1.4 ± 1.8 and 3.3 ± 3.6 for KBT and FCM respectively, so to neutralize these amounts of fetal whole blood we need administering RhIG from 50 to 300 μg and multiples of these doses.

Administration of 100 IU (20 μg) Rh D immunoglobulin has been demonstrated to protect against 1 ml of fetal red cells, 500 IU (100 μg) should protect against FMH of up to 5 ml of fetal red cells and 1,500 IU (300 μg) Rh D immunoglobulin against FMH of approximately 15 ml of fetal red cells [20]. Before 20 weeks’ gestation 250 IU should be given. After 20 weeks’ gestation blood should be taken at least for the conventional KBT to estimate the size of the FMH and 500 IU of RhIG given [14]. This showed for the FMH we have calculated in the current study, 500 IU (100 μg) dose of RhIG would have been sufficient for 92.5% and 87% of the 39 and 45 Rh D-negative mothers if KBT and FCM were employed, respectively. This result was consistent with a study conducted by Lubusky *et al.* that revealed during normal vaginal delivery as well as during delivery by cesarean section, FMH of less than 5 mL occurs in the great majority of cases, and thus for the prevention of D alloimmunization, RhIG dose of 100 μg should be sufficient [21].

The widespread adoption of postpartum immunoprophylaxis with a single dose of Rh D immunoglobulin dramatically reduced the incidence of Rh D immunization, and HDFN. However, despite this the incidence of Rh D immunisation during pregnancy remains at approximately 1-2%. This can partly be explained by the occurrence of a FMH of a volume larger than the protection offered by a single dose of Rh D Immunoglobulin [22]. In our study, 1.3% and 2.7% of FMH calculated were >30 mL as quantified by KBT and FCM methods, respectively that requires a neutralizing dose of more than 300 μg RhIG.

On the other hand our result was inconsistent with Johnson e*t al.* findings that stated in only 0.5% of deliveries FMH exceeds 25 mL. It is, therefore, important that the volume of FMH is accurately assessed so that, if necessary, a supplementary dose(s) of RhIG can be administered and maternal alloimmunisation prevented [14].

### Risk factors

The result of our study has shown all expected risk factor were not associated with FMH, but gestational age 20–28 weeks was significantly different from other gestational ages by one-way ANOVA with mean and standard deviation (M = 10.9 SD = 11.1) (Figure 2). This could be because of the fact that most of our participants at this gestation age were having abortion or miscarriage of delivery. This study was consistent with the study conducted by Von Stein et al. which demonstrates an increased incidence of FMH in patients threaten to abortion compared with a gestationally matched control group [23].

Our results also consistent with the study conducted by Salim et al. that revealed there appears to be no difference in the incidence of large fetomaternal hemorrhage between cesarean and vaginal deliveries or between singleton and multiple deliveries [24]. Again Pelikan et al. reported no difference between vaginal and cesarean deliveries [25]. Besides this, David et al. identified Twin pregnancy as the only independent risk factor for severe fetal-to-maternal transfusion, but ABO-incompatibility between mother and infant seems to be protective against Rh D-alloimmunization [26].

Our result was inconsistent with a study conducted by Lubusky M et al. that verified delivery by cesarean section presented a higher risk of incidence of FMH of more than 2.5 mL (odds ratio, 2.2; p = 0.004) when compared with normal vaginal delivery. It did not, however, present a significant risk factor for the incidence of excessive volumes of FMH of more than 5 mL [21]. We thought our study did not demonstrate associations with many expected risk factors because of the smaller sample size we used than many studies conducted with this title/area.

This study is sounder if it were conducted with more sample size and using anti-HgF monoclonal antibody besides the two methods.

## Conclusions

**In conclusion,** FMH has been detected in 52% and 60% of our participants by KBT and FCM method, respectively. The amount of FMH calculated was <10 mL fetal blood in 92.5% and 87% of FMH cases as quantified by KBT and FCM methods, respectively. This indicated most of the FMH calculated could have been neutralized by lower doses which might have incurred lower costs than the 300 μg dose, the only available dose in Ethiopia as well unaffordable by 28% of our participants.

Conversely, large FMH (>30 ml) occurred in 1.3% and 2.7% of FMH cases calculated by KBT and FCM methods ,respectively, which require a neutralizing dose of more than 300 μg RhIG indicating the need for FMH calculation to ensure a sufficient dose of RhIG given for RhD negative mothers. The correlations between two methods was good (r = 0.828 and 0.897) for categorized and continuous values, respectively, (p = 0.000 for both) with moderate agreement.

**Based on this study,** further investigation into the cost-effectiveness and scalability of patient-specific dosing of prophylactic anti-D is warranted. Developing of optimized testing (KBT and/or FC) and accessing dosing protocols is needed in health facilities.

## References

[1] Greer JP, Foerster J, Lukens J, Rodgers G, Paraskevas F, Glader B, Neff A (2003). Autoimmune Hemolytic Anemia. Wintrobe’s Clinical Hematology.

[2] AABB (2005). Technical Manual.

[3] Pourazar A, Homayouni A, Rezaei A, Andalib A, Oreizi F (2008). The Assessment of Feto-Maternal Hemorrhage in an Artificial Model Using Anti-D and Anti-Fetal Hemoglobin Antibody by FCM, Iran. Biomed J.

[4] Lafferty JD, Raby A, Crawford L, Linkins LA, Richardson H, Crowther M (2003). Fetal-Maternal Hemorrhage Detection in Ontario. Am J Clin Pathol.

[5] Blood BCSH (1999). Transfusion and General Haematology Task Forces. The estimation of fetomaternal haemorrhage, GUIDELINES. Transfus Med.

[6] Quinley E (1998). Immunohematology, principles and practice.

[7] Judd WJ (2001). Practice guidelines for prenatal and perinatal immunohematology, revisited. Transfusion.

[8] Kim YA, Makar RS (2012). Detection of fetomaternal hemorrhage. AJH.

[9] Dziegiel MH, Nielsen LK, Berkowicz A (2006). Detecting fetomaternal hemorrhage by FCM. Transfus Med.

[10] Koelewijn J, de Haas M, Vrijkotte T, van der Schoot C, Bonsel G (2009). Risk factors for RhD immunization despite antenatal and postnatal anti-D prophylaxis. BJOG.

[11] Wylie BJ, D’Alton ME (2010). Fetomaternal Hemorrhage. Obstet Gynecol.

[12] Lubusky M (2010). Prevention of RhD alloimmunization in RhD negative women. Biomed Pap Med Fac Univ Palacky Olomouc Czech Repub.

[13] Osaro E, Charles AT (2010). Rh isoimmunization in Sub-Saharan Africa indicates need for universal access to anti-RhD immunoglobulin and effective management of D-negative pregnancies. Int J of Women’s Health.

[14] Austin E, Bates S, Silva M, Howarth D, Lubenko A, Rowley M, Scott M, Thomas E, White J, Williams M, BCSH Blood Transfusion and General Haematology Task Forces (2009). The estimation of fetomaternal haemorrhage, Guidelines.

[15] Augustson BM, Fong EA, Grey DE, Davies JI, Erber WN (2006). Postpartum anti-D: can we safely reduce the dose?. MJA.

[16] Johnson PRE, Tait RC, Austin EB, Shwe KH, Lee D (1995). Flow cytometry in diagnosis and management of large fetomaternal haemorrhage. J Clin Pathol.

[17] Pastoret C, Le PJ, Fest T, Roussel M (2013). Evaluation of FMH QuikQuant for the detection and quantification of fetomaternal hemorrhage. Cytometry Part B.

[18] Savithrisowmya S, Singh M, Kriplani A, Agarwal N, Mehra NK, Bhatla N (2008). Fetomaternal Hemorrhage by Flow Cytometry and Kleihauer-Betke Test in Rh-Negative Pregnancies. Gynecol Obstet Invest.

[19] Fung K, Fung K, Eason E (2003). Prevention of Rh alloimmunization. SOGC clinical Practice Guidelines. J Obstet Gynaecol Can.

[20] National Health and Medical Research Council (2003). Guidelines on the prophylactic use of Rh D immunoglobulin (anti-D) in obstetrics.

[21] Lubusky M, Simetka O, Studnickova M, Prochazka M, Ordeltova M, Vomackova K (2012). Fetomaternal hemorrhage in normal vaginal delivery and in delivery by cesarean section. Transfusion.

[22] ANZSBT (2003). Guidelines for Laboratory Assessment of Fetomaternal Haemorrhage. Australian and New Zealand Society of Blood Transfusion, Sydney.

[23] Von stein GA, Munsick RA, Stiver K, Ryder K (1992). Fetomaternal Hemorrhage in Threaten Abortion. Obstet Gynecol.

[24] Salim R, Ben-Shlomo I, Nachum Z, Mader R, Shalev E (2005). The Incidence of Large Fetomaternal Hemorrhage and the Kleihauer-Betke Test. Obstet Gynecol.

[25] Pelikan DM, Scherjon SA, Mesker WE, de Groot-Swings GM, Brouwer-Mandema GG, Tanke HJ, Kanhai HH (2004). Quantification of fetomaternal hemorrhage: a comparative study of the manual and automated microscopic Kleihauer-Betke tests and FCM in clinical samples. JOGC.

[26] David M, Smidt J, Chen FCK, Stein U, Dudenhausen JW (2005). Risk factors for fetal maternal transfusion in Rh D-negative women results of a prospective study on 942 pregnant women. J Perinat Med.

[CR27] The pre-publication history for this paper can be accessed here:http://www.biomedcentral.com/1471-2393/14/358/prepub

